# Difference in the Prognostic Significance of N-Terminal Pro-B-Type Natriuretic Peptide between Cardioembolic and Noncardioembolic Ischemic Strokes

**DOI:** 10.1155/2015/597570

**Published:** 2015-03-05

**Authors:** Seung-Jae Lee, Dong-Geun Lee, Dal-Soo Lim, Sukkeun Hong, Jin-Sik Park

**Affiliations:** ^1^Department of Neurology, Sejong General Hospital, Bucheon 422-711, Republic of Korea; ^2^Department of Cardiology, Sejong General Hospital, Bucheon 422-711, Republic of Korea

## Abstract

*Background and Aim*. Because B-type natriuretic peptide is a powerful predictor of heart failure, its capability to predict a fatal outcome in stroke might be limited to the cardioembolic stroke subtype. In this study, we attempt to ascertain the difference in the prognostic value of N-terminal pro-B-type natriuretic peptide (NT-proBNP) between cardioembolic and noncardioembolic stroke subgroups. *Methods*. 410 acute stroke patients were included. According to the presence of a cardioembolic source (CES), there were 221 patients with CES and 189 patients without CES. Logistic regression analysis was performed to ascertain the association between NT-proBNP and 6-month mortality/functional outcome in each group. *Results*. The mean age of our patients was 67.2 years (range, 18–97 years). NT-proBNP was a multivariate independent predictor of mortality in the CES group alone, whereas it was only a univariate predictor of 6-month mortality in the total patient and non-CES groups with its association disappearing in the multivariate model. In addition, it was only a univariate predictor of good functional outcome in all of the groups. *Conclusions*. Our data suggest that NT-proBNP can more reliably predict 6-month mortality in patients with cardioembolic stroke than in patients with other stroke subtypes.

## 1. Introduction

It has been well established that B-type natriuretic peptide (BNP) is a marker of poor outcome in patients with ischemic stroke [1–7]. However, it remains to be clarified whether this finding can be applied to all subtypes of stroke. Because BNP is a powerful predictor of heart failure, especially left ventricular (LV) systolic dysfunction [8], its capability to predict a fatal outcome in stroke might be only due to its prognostic role in heart disease. Therefore, the classification of stroke patients according to the presence of cardioembolic stroke mechanism and subsequent analysis of the prognostic role of BNP in each group can be useful in determining the clinical significance of BNP in predicting stroke outcome.

Accordingly, we classified our patients into two groups, with and without a cardioembolic source (CES), and then evaluated the prognostic value of N-terminal proBNP (NT-proBNP) in each stroke group.

## 2. Methods

With the approval of Institutional Review Board of Sejong General Hospital, we initially studied a consecutive cohort of 465 patients with acute ischemic stroke or transient ischemic attack with a lesion, who were admitted to the neurovascular or cardiovascular center of our hospital within 7 days after stroke onset between January 2011 and January 2014. From these patients, we excluded 49 patients with incomplete study data (no data of cardiac evaluation or NT-proBNP) and 6 patients who did not have the 6-month outcome data, and finally a total of 410 patients were included in this analysis.

The study patients were classified into two groups according to the presence of a CES, based on cardiac evaluation including electrocardiography/24-hour Holter monitoring and echocardiography. Transthoracic echocardiography was performed in the 410 included patients, while transesophageal echocardiography was performed in only 79 patients (19.3%) who had a high probability of intracardiac thrombus and were able to accept an esophageal transducer. Based on the previous articles [9–11], CESs were classified as major and minor sources. Major CES included atrial fibrillation/flutter (AF, 143 patients), mitral stenosis (14 patients), left atrial thrombus (12 patients), mechanical prosthetic valve (17 patients), dilated cardiomyopathy (6 patients), sick sinus syndrome (6 patients), myocardial infarction (MI) within 4 weeks (12 patients), LV thrombus (5 patients), akinetic LV segment or aneurysm (46 patients), atrial myxoma (1 patient), and infective endocarditis (4 patients). Minor CES included mitral valve prolapse or regurgitation (36 patients), mitral annular calcification (20 patients), spontaneous echo contrast (31 patients), atrial septal aneurysm (1 patient), patent foramen ovale or atrial septal defect (23 patients), bioprosthetic valve (5 patients), LV systolic dysfunction (ejection fraction < 40%; 51 patients), hypokinetic LV segment (11 patients), and MI between 4 weeks and 6 months (1 patient). Finally, 221 (53.9%) and 189 (46.1%) patients were assigned to the different stroke groups with and without CES, respectively.

The clinical information included age, gender, waist circumference (WC; measured at the highest point of the iliac crest at minimal respiration while patients were in the supine position), history of hypertension (defined as the use of an antihypertensive agent before admission, or systolic pressure >140 mmHg, or diastolic pressure >90 mmHg demonstrated on repeated examinations at least one month after presentation with stroke), diabetes mellitus (defined as a fasting blood glucose level >126 mg/dL or a history of being treated for diabetes mellitus), and hyperlipidemia (defined as a total cholesterol level >200 mg/dL or a low density lipoprotein cholesterol >130 mg/dL at the time of presentation or a history of treatment). In addition, current cigarette smoking, heavy alcohol consumption (>26 bottle drinks/month of Soju; about 20% alcohol), a previous history of stroke, and ischemic heart disease (defined as a known history or clinical demonstration of MI or angina pectoris) were noted.

Blood samples for the analysis of NT-proBNP were collected in heparin-containing tubes within 24 hours after admission and then centrifuged at 4°C for 7 minutes at 3000 g. NT-proBNP levels were determined by electrochemiluminescence in an automated analyzer (Cobas e 602; Roche Diagnostics, Mannheim, Germany).

Statistical analyses were performed with SPSS software, version 18.0 (SPSS Inc., Chicago, IL). The independent* t*-test or chi-square test was used to compare the difference between the two groups. Univariate and multivariate linear regression analyses were performed to identify the variables associated with NT-proBNP level in the total 410 patients. Univariate logistic regression analysis was performed to ascertain the association between independent variables and 6-month mortality/good functional outcome (modified Rankin scale ≤2) in the total patient, CES, and non-CES groups. The independent variables included age, gender, hypertension, diabetes mellitus, hyperlipidemia, ischemic heart disease, current smoking, heavy alcohol use, history of previous stroke, AF, previous antithrombotic use, previous statin therapy, LV systolic dysfunction (ejection fraction < 40%), the presence of any CES, NT-proBNP ≥the median levels in each group (378.2 pg/mL in the total patient group; 1018.0 pg/mL in the CES group; 98.6 pg/mL in the non-CES group), creatinine (mg/dL), and National Institutes of Health Stroke Scale (NIHSS) score at admission. However, some variables were unavoidably excluded from the analysis of CES and non-CES groups because of the characteristics of each group; that is, AF, LV systolic dysfunction, and the presence of a CES were excluded from the analysis of the non-CES group, and the presence of a CES was excluded from the analysis of the CES group. Then, multivariate logistic regression analysis (using the variables with *P* < 0.2 on the univariate model) was performed to identify the variables independently associated with 6-month mortality and good functional outcome. Unadjusted and adjusted odds ratio (OR) and 95% confidence interval (CI) were obtained.* P* values < 0.05 were considered statistically significant.

## 3. Results

The mean age of the 410 patients included in this study (212 males and 198 females) was 67.2 years (range, 18–97 years) at admission. As shown in Table 1, the non-CES group had a significantly higher frequency of hyperlipidemia, smoking, and heavy alcohol use, while the CES group had a significantly higher prevalence of previous antithrombotic therapy and ischemic heart disease. In addition, the level of NT-proBNP and initial NIHSS score were significantly increased in patients with CES.

The linear regression analysis showed that NT-proBNP level was positively correlated with age (*P* = 0.003), female gender (*P* = 0.006), the presence of a CES (*P* < 0.001), LV systolic dysfunction (*P* < 0.001), creatinine (*P* < 0.001), and NIHSS score (*P* < 0.001) and was negatively correlated with WC (*P* < 0.001). The multivariate model using the above variables except for LV systolic dysfunction indicated that female gender, the presence of a CES, creatinine, NIHSS score, and WC were significantly associated with NT-proBNP level.

In the univariate analysis of mortality in the total patient group, age (OR 1.045; 95% CI 1.016–1.074), female gender (OR 2.163; 95% 1.118–4.185), ischemic heart disease (OR 2.273; 95% CI 1.153–4.482), LV systolic dysfunction (OR 4.486; 2.048–9.823), CES (OR 2.740; 95% CI 1.340–5.600), NT-proBNP (OR 16.323; 95% CI 4.960–53.717), creatinine (OR 1.408; 95% CI 1.153–1.720), and NIHSS score (OR 1.160; 95% CI 1.114–1.208) were significantly associated with 6-month mortality. Among these variables, LV systolic dysfunction (OR 3.818; 95% CI 1.436–10.151), creatinine (OR 1.566; 95% CI 1.118–2.193), and NIHSS score (OR 1.135; 95% CI 1.078–1.195) remained significant predictors of mortality in the multivariate model. The univariate model of good functional outcome showed that age (OR 0.948; 95% CI 0.931–0.966), female gender (OR 0.368; 95% CI 0.237–0.570), smoking (OR 2.836; 95% CI 1.642–4.899), alcohol (OR 2.843; 95% CI 1.441–5.612), LV systolic dysfunction (OR 0.359; 95% CI 0.189–0.682), NT-proBNP (OR 0.299; 95% CI 0.190–0.469), and NIHSS score (OR 0.799; 95% CI 0.764–0.836) significantly predicted the 6-month functional outcome. However, NIHSS score (OR 0.791; 95% CI 0.742–0.844) alone remained a significant predictor in the multivariate model (Table 2).

In the analysis of the CES group, age (OR 1.036; 95% CI 1.004–1.069), LV systolic dysfunction (OR 4.400; 95% CI 2.009–9.638), NT-proBNP (OR 12.614; 95% CI 3.713–42.853), creatinine (OR 1.464; 95% CI 1.116–1.920), and NIHSS score (OR 1.131; 95% CI 1.082–1.182) were associated with mortality in the univariate model. Subsequent multivariate analysis demonstrated that NT-proBNP (OR 4.297; 95% CI 1.073–17.203), together with LV systolic dysfunction (OR 2.963; 95% CI 1.068–8.220), creatinine (OR 1.489; 95% CI 1.063–2.085), and NIHSS score (OR 1.125; 95% CI 1.067–1.185), was independently predictive of mortality. However, NT-proBNP (OR 0.199; 95% CI 0.106–0.373), along with age (OR 0.958; 95% CI 0.936–0.981), female gender (OR 0.486; OR 0.273–0.863), and LV systolic dysfunction (OR 0.367; 95% CI 0.193–0.699), was only a univariate predictor of functional outcome; NIHSS score (OR 0.802; 95% CI 0.754–0.854) alone proved to be the multivariate predictor of functional outcome in this group (Table 3).

In the analysis of the non-CES group, among age (OR 1.067; 95% CI 1.007–1.130), NT-proBNP (OR 12.458; 95% CI 1.575–98.558), and NIHSS score (OR 1.230; 95% CI 1.123–1.346) which were significantly associated with mortality in the univariate model, NIHSS score (OR 1.211; 95% CI 1.083–1.354) alone was independently predictive of mortality in the multivariate model. With respect to 6-month functional outcome, age (OR 0.935; 95% CI 0.908–0.964), female gender (OR 0.260; 95% CI 0.131–0.516), smoking (OR 3.697; 95% CI 1.670–8.182), alcohol use (OR 2.687; 95% CI 1.168–6.185), NT-proBNP (OR 0.302; 95% CI 0.151–0.603), and NIHSS score (OR 0.751; 95% CI 0.682–0.828) were significantly predictive in the univariate model, but only age (OR 0.946; 95% CI 0.905–0.989) and NIHSS score (OR 0.761; 95% CI 0.679–0.853) were the multivariate predictors (Table 4).

## 4. Discussion

BNP is mainly released from the cardiac wall in response to excessive myocardial stretch and is thus a traditional biomarker of heart failure [8]. Recently in the field of stroke research, many studies have shown that BNP can be a powerful predictor of poor outcome, especially mortality [1–7]. However, the mechanism underlying the capability of BNP to predict mortality is unclear, but it is principally presumed to be related to the fact that BNP is well correlated with global cardiac function. Specifically, the BNP level does not only reflect the degree of LV dysfunction [8, 12], but it also correlates with left atrial dysfunction, predicting the risk of thromboembolism in patients with AF [13, 14]. In addition, it is known to be correlated with NIHSS score, which is proportional to the infarct size [2, 15, 16]. Besides, it has been associated with other adverse conditions such as advanced age and renal dysfunction [12]. Our results also showed that NT-proBNP was significantly associated with age, NIHSS score, creatinine, and adverse heart conditions including reduced LV ejection fraction (<40%) and the presence of a CES.

However, it is not clear whether the prognostic value of BNP differs according to the stroke subtype, specifically according to the presence of cardioembolic stroke mechanism. Because the BNP level is assumed to be principally dependent on the cardiac condition, its capability to predict stroke outcome may be limited to cardioembolic stroke. In addition, most of the studies to date did not categorize the study patients according to stroke classification for estimating the prognostic value of BNP in the prediction of stroke outcome [2–4]. Although some studies included stroke subtype as a variable in the analysis, they did not evaluate the role of BNP in each subtype [5, 6].

Recently, a study analyzed the prognostic role of BNP in each outcome in the cardioembolic and noncardioembolic stroke subtypes. The results of the study showed that BNP can independently predict the prognosis of cardioembolic stroke, but not that of noncardioembolic stroke [1]. Our data also showed that NT-proBNP was a multivariate independent predictor of mortality in the CES group alone, whereas it was only a univariate predictor of mortality in the total patient and non-CES groups with its association with 6-month mortality disappearing in the multivariate model. In addition, it was only a univariate predictor of good functional outcome in all of the groups. Actually, NIHSS score was the most consistent univariate and multivariate predictor of both mortality and functional outcome in our patients.

Accordingly, the results of this study may suggest that the prognostic role of NT-proBNP in predicting mortality can be more reliably applied to the cardioembolic stroke subtype than the other stroke subtypes. However, this finding should be cautiously applied to the general stroke population until additional large-scale studies on this topic are performed in the future. This is mainly because of some limitations of this study. First, this study was based on single-center stroke registry data. In addition, infarct volume was not included in our analysis although NIHSS score, known to be well correlated with infarct volume, was used in the analysis. Finally, we measured the plasma level of NT-proBNP only once and thus could not consider the serial changes in the level.

## 5. Conclusions

Our result indicates that the plasma level of NT-proBNP can more reliably predict 6-month mortality in patients with cardioembolic stroke than in patients with the other stroke subtypes. Further studies are needed to clarify the prognostic role of BNP in each stroke subtype.

## Figures and Tables

**Table 1:** General characteristics of the study population; mean ± SD, *n* (%).

	Cardioembolic source		*P*
	Present *N* = 221	Absent *N* = 189	
Age, yr	67.7 ± 14.2	66.5 ± 13.6	0.401
Female gender	113 (51.1)	85 (45.0)	0.214
Hypertension	145 (65.6)	139 (73.5)	0.083
Diabetes	60 (27.1)	67 (35.4)	0.070
Hyperlipidemia	113 (51.1)	116 (61.4)	0.037
Current smoking	46 (20.8)	70 (37.0)	<0.001
Alcohol use	19 (8.6)	54 (28.6)	<0.001
Previous stroke	41 (18.6)	24 (12.7)	0.106
Ischemic heart disease	65 (29.4)	20 (10.6)	<0.001
Antiplatelet	107 (48.4)	59 (31.2)	<0.001
Warfarin	58 (26.2)	0 (0)	<0.001
Creatinine	1.14 ± 1.06	1.01 ± 1.09	0.221
NT-proBNP	3710.1 ± 7715.1	518.5 ± 2232.2	<0.001
NIHSS at admission	7.5 ± 8.8	4.7 ± 5.6	<0.001

NT-proBNP: N-terminal pro-B-type natriuretic peptide; NIHSS: National Institutes of Health Stroke Scale.

**Table 2:** Univariate and multivariate predictors of mortality and functional outcome in the total patient group; odds ratio (95% confidence interval).

Variables	Mortality		Functional outcome	
	Univariate	Multivariate	Univariate	Multivariate
Age	1.045 (1.016–1.074)		0.948 (0.931–0.966)	
Female gender	2.163 (1.118–4.185)		0.368 (0.237–0.570)	
Ischemic heart disease	2.273 (1.153–4.482)			
Current smoking			2.836 (1.642–4.899)	
Heavy alcohol use			2.843 (1.441–5.612)	
LV systolic dysfunction	4.486 (2.048–9.823)	3.818 (1.436–10.151)	0.359 (0.189–0.682)	
Cardioembolic source	2.740 (1.340–5.600)			
NT-proBNP	16.323 (4.960–53.717)		0.299 (0.190–0.469)	
Creatinine	1.408 (1.153–1.720)	1.566 (1.118–2.193)		
NIHSS at admission	1.160 (1.114–1.208)	1.135 (1.078–1.195)	0.799 (0.764–0.836)	0.791 (0.742–0.844)

LV: left ventricular; NT-proBNP: N-terminal pro-B-type natriuretic peptide; NIHSS: National Institutes of Health Stroke Scale.

**Table 3:** Univariate and multivariate predictors of mortality and functional outcome in patients with a cardioembolic source; odds ratio (95% confidence interval).

Variables	Mortality		Functional outcome	
	Univariate	Multivariate	Univariate	Multivariate
Age	1.036 (1.004–1.069)		0.958 (0.936–0.981)	
Female gender			0.486 (0.273–0.863)	
LV systolic dysfunction	4.400 (2.009–9.638)	2.963 (1.068–8.220)	0.367 (0.193–0.699)	
NT-proBNP	12.614 (3.713–42.853)	4.297 (1.073–17.203)	0.199 (0.106–0.373)	
Creatinine	1.464 (1.116–1.920)	1.489 (1.063–2.085)		
NIHSS at admission	1.131 (1.082–1.182)	1.125 (1.067–1.185)	0.810 (0.769–0.854)	0.802 (0.754–0.854)

LV: left ventricular; NT-proBNP: N-terminal pro-B-type natriuretic peptide; NIHSS: National Institutes of Health Stroke Scale.

**Table 4:** Univariate and multivariate predictors of mortality and functional outcome in patients without a cardioembolic source; odds ratio (95% confidence interval).

Variables	Mortality		Functional outcome	
	Univariate	Multivariate	Univariate	Multivariate
Age	1.067 (1.007–1.130)		0.935 (0.908–0.964)	0.946 (0.905–0.989)
Female gender			0.260 (0.131–0.516)	
Current smoking			3.697 (1.670–8.182)	
Heavy alcohol use			2.687 (1.168–6.185)	
NT-proBNP	12.458 (1.575–98.558)		0.302 (0.151–0.603)	
NIHSS at admission	1.230 (1.123–1.346)	1.211 (1.083–1.354)	0.751 (0.682–0.828)	0.761 (0.679–0.853)

NT-proBNP: N-terminal pro-B-type natriuretic peptide; NIHSS: National Institutes of Health Stroke Scale.

## References

[1] Rost N. S., Biffi A., Cloonan L. (2012). Brain natriuretic peptide predicts functional outcome in ischemic stroke. *Stroke*.

[2] Mäkikallio A. M., Mäkikallio T. H., Korpelainen J. T. (2005). Natriuretic peptides and mortality after stroke. *Stroke*.

[3] Jensen J. K., Atar D., Kristensen S. R., Mickley H., Januzzi J. L. (2009). Usefulness of natriuretic peptide testing for long-term risk assessment following acute ischemic stroke. *The American Journal of Cardiology*.

[4] Sharma J. C., Ananda K., Ross I., Hill R., Vassallo M. (2006). N-terminal proBrain natriuretic peptide levels predict short-term poststroke survival. *Journal of Stroke and Cerebrovascular Diseases*.

[5] Chang L., Yan H., Li H. (2014). N-terminal probrain natriuretic peptide levels as a predictor of functional outcomes in patients with ischemic stroke. *NeuroReport*.

[6] Shibazaki K., Kimura K., Okada Y. (2009). Plasma brain natriuretic peptide as an independent predictor of in-hospital mortality after acute ischemic stroke. *Internal Medicine*.

[7] García-Berrocoso T., Giralt D., Bustamante A. (2013). B-Type natriuretic peptides and mortality after stroke a systematic review and meta-analysis. *Neurology*.

[8] Reddy P., Samson R. (2013). Clinical utility of natriuretic peptides in left ventricular failure. *Southern Medical Journal*.

[9] Adams H. P., Bendixen B. H., Kappelle L. J. (1993). Classification of subtype of acute ischemic stroke. Definitions for use in a multicenter clinical trial. TOAST. Trial of Org 10172 in Acute Stroke Treatment. *Stroke*.

[10] Arboix A., Alió J. (2012). Acute cardioembolic cerebral infarction: answers to clinical questions. *Current Cardiology Reviews*.

[11] Weir N. U. (2008). An update on cardioembolicstroke. *Postgraduate Medical Journal*.

[12] Daniels L. B., Maisel A. S. (2007). Natriuretic peptides. *Journal of the American College of Cardiology*.

[13] Okada Y., Shibazaki K., Kimura K. (2011). Brain natriuretic peptide is a marker associated with thrombus in stroke patients with atrial fibrillation. *Journal of the Neurological Sciences*.

[14] Roldán V., Vílchez J. A., Manzano-Fernández S. (2014). Usefulness of N-terminal pro-B-type natriuretic Peptide levels for stroke risk prediction in anticoagulated patients with atrial fibrillation. *Stroke*.

[15] Nakagawa K., Yamaguchi T., Seida M. (2005). Plasma concentrations of brain natriuretic peptide in patients with acute ischemic stroke. *Cerebrovascular Diseases*.

[16] Nogami M., Shiga J., Takatsu A., Endo N., Ishiyama I. (2001). Immunohistochemistry of atrial natriuretic peptide in brain infarction. *Histochemical Journal*.

